# REFERQUAL: a pilot study of a new service quality assessment instrument in the GP exercise referral scheme setting

**DOI:** 10.1186/1472-6963-6-61

**Published:** 2006-05-25

**Authors:** Don Cock, Iain C Adams, Adrian B Ibbetson, Phil Baugh

**Affiliations:** 1 Centre for Sport and Dance Studies, Liverpool John Moores University, Liverpool, UK; 2 Department of Tourism and Leisure Management, University of Central Lancashire, Preston, UK; 3 Department of Information and Finance, University of Central Lancashire, Preston, UK

## Abstract

**Background:**

The development of an instrument accurately assessing service quality in the GP Exercise Referral Scheme (ERS) industry could potentially inform scheme organisers of the factors that affect adherence rates leading to the implementation of strategic interventions aimed at reducing client drop-out.

**Methods:**

A modified version of the SERVQUAL instrument was designed for use in the ERS setting and subsequently piloted amongst 27 ERS clients.

**Results:**

Test re-test correlations were calculated via Pearson's 'r' or Spearman's 'rho', depending on whether the variables were Normally Distributed, to show a significant (mean r = 0.957, SD = 0.02, p < 0.05; mean rho = 0.934, SD = 0.03, p < 0.05) relationship between all items within the questionnaire. In addition, satisfactory internal consistency was demonstrated via Cronbach's 'α'. Furthermore, clients responded favourably towards the usability, wording and applicability of the instrument's items.

**Conclusion:**

REFERQUAL is considered to represent promise as a suitable tool for future evaluation of service quality within the ERS community. Future research should further assess the validity and reliability of this instrument through the use of a confirmatory factor analysis to scrutinise the proposed dimensional structure.

## Background

Exercise Referral Schemes (ERSs) in the UK involve a recommendation from a clinician to a patient concerning the up-take of physical activity, based upon certain pre-determined criteria. The patient then attends a local exercise centre, where an advanced exercise instructor will create an appropriate individualised programme, usually designed to last around 12–15 weeks. The publication of 'Exercise Referral Systems: A National Quality Assurance Framework' [1] was, in part, aimed at improving standards among existing ERSs and aiding the development of new systems. However, many schemes both pre- and post-publication of this document suffered from and struggled with issues relating to poor levels of adherence [2,3]. Client perceptions of excellent service quality are highlighted as being crucial to the process of attracting new members and retaining existing members in the leisure management industry [4], a theme not uncommon within other service industries [5-7]. The development of a tool to accurately assess service quality within the Exercise Referral setting, could, therefore, have a considerable impact on informing ERS co-ordinators of the factors that affect adherence and subsequent enable the establishment of strategic interventions to reduce the likelihood of client drop-out. The concept of service quality has been the subject of considerably lively academic debate for two decades, since being brought to the fore by Parasuraman, Zeithaml and Berry (PZB) [8,9] in SERVQUAL [see Additional File 1], a multiple-item scale aimed at the measurement of service quality, structured around five dimensions:

• Tangibles: Physical facilities, equipment, and appearance of personnel.

• Reliability: Ability to perform the promised service dependably and accurately.

• Responsiveness: Willingness to help customers and provide prompt service.

• Assurance: Knowledge and courtesy of employees and their ability to inspire trust and confidence.

• Empathy: Caring, individualised attention the firm provides its customers.

[9]

The SERVQUAL instrument comprises twenty two questions tailored to assess customers' expectation of service quality, followed by a further twenty two items designed to identify the clients' perception of the same issues. Each item is scored on a seven point Likert scale, thus allowing the equation Q = P - E to be calculated [9].

Since PZB's [9] publication, SERVQUAL has been the target of substantial scrutiny whilst also dominating service quality assessment [10-15], being applied to numerous settings, including health [16-20] but not to ERSs. A considerable part of the academic debate over the past two decades has discussed the conceptual accuracy of SERVQUAL's perception minus expectation equation and whether performance-only measures would be more appropriate [21,22]. This discussion is beyond the scope of this paper; however, it should be noted that in settings such as ERSs, where the perceptions of the absent client are as valuable (and possibly even more valuable) as the life-long adherent, performance-only measures are of no use [21,22]. Furthermore, Sureshchandar *et al*. [23] go as far as to say there is "universal conformity that the twenty two items [of SERVQUAL] are reasonably good predictors of service quality in its entirety" (p. 2).

Applying SERVQUAL in its pure form (*i.e*. without modification) to any service is widely criticised in the literature [14,24]. However, tailoring the instrument to a specific setting by adding additional items or modifying existing questions to supplement knowledge and understanding, SERVQUAL can give a unique insight into the service quality [25]. PZB [8,9] confirm adaptations to SERVQUAL are necessary if an accurate measure of service quality is to be established across a diverse range of industries.

## Methods

### Creation of REFERQUAL

Tailoring and augmenting the SERVQUAL instrument has been identified as a necessary requirement when being applied to a new service industry [25]. A client adherence classification structure was created in conjunction with several ERS organisers. The structure is self-reporting, exhaustive and mutually exclusive. The five statements are constructed to classify clients relating to the extent of adherence to the referral process:

A Referred clients who did not attend at all.

B Clients who started, but dropped out before completion.

C Clients attending, but had not completed the referral at the time of receiving the questionnaire.

D Clients who completed the referral but did not continue exercising.

E Clients who are still exercising having completed referral.

In addition to the self-reporting adherence criteria, clients were also requested to complete certain demographic information addressing factors raised by the literature such as gender, age, occupation [26-29] and marital status [30,31]. Two further questions emanating from the literature were included at the beginning of the instrument as demographic issues due to the nature of the items containing no element of expectation and so could not be included as a perception minus expectation item:

'Do you consider yourself to be physically active whilst carrying out the duties demanded by your occupation?'

'Was reducing weight one of the reasons you were referred to the scheme?'

An adaptation of the Blair *et al*. [32] 7-day Physical Activity Recall (7PAR) questionnaire was also included in REFERQUAL to assess the exercise level of participants outside any occupational demand. The inclusion of the adapted 7PAR was aimed at offering the potential for differentiation between those participants who were sufficiently physically active and those who were not irrespective of adherence grouping. Furthermore, respondents were invited to report the location of the physical activity (*i.e*. leisure centre or elsewhere), offering the researcher greater insight into whether the service quality of the operational aspects of the scheme or the overall management of the referral site may have most critically affected adherence.

Considering the lengthy and relatively complex nature of SERVQUAL, the 7PAR was simplified from five to three levels of physical activity (light, moderate and vigorous), although duration of exercise was retained. Definitions and examples of light, moderate and vigorous physical activity were given.

Many client-related factors established as being significant determinants to adherence in the literature, formed the foundation from which tailoring of the SERVQUAL instrument was undertaken. Part of the tailoring of the SERVQUAL instrument involved the creation of two new dimensions as a number of topics raised in the literature did not conceptually 'fit' into the existing dimensional framework. The first of these relates to the relationship between the client and the GP – an association distinct to the ERS industry from any previously examined. The second relates to personal perceptions of exercise. The existing 22 items of SERVQUAL were supplemented with the questions [see Additional File 2].

Modification of SERVQUAL to eliminate negatively-worded questions included in the original instrument has received unanimous support, this was also taken into account when tailoring the items to the ERS Setting [11].

### Piloting methods

The pre-pilot REFERQUAL appended with a feedback sheet was distributed to six attendees at five ERSs and also to the corresponding scheme organisers. The five ERSs had been selected to participate in the main post-pilot study. Feedback was received with respect to the instrument's ease of use, wording and any other topic the respondents felt relevant. Some questions were subsequently removed the instrument whilst others were modified following feedback to enhance applicability and clarity or to eliminate duplicity.

Subsequently, one exercise class comprising 30 clients was invited to participate in the pilot study. The Exercise Professional leading the group distributed REFERQUAL by hand and responses were completed whilst the exercisers were at the centre. The Exercise Professional subsequently repeated the operation two weeks later with the same group. Twenty-seven of the 30 in the group completed both questionnaires, a response rate of 90%, the other three participants were absent for one of the two sessions. Again, respondents were also invited to comment on the instruments ease of use, wording and any general concerns relating to REFERQUAL.

### Analysis

In order to determine test re-test reliability, correlation will be calculated via Pearson's 'r' or Spearman's 'rho' depending on the distribution of the individual items. One sample Kolmogorov-Smirnov (K-S) tests will be used to determine whether items are drawn from normally distributed data. K-S testing is a suitable measure of distribution regardless of sample size [33], is consistent against all alternatives [34] and frequently outperforms other measures [35]. Internal Consistency will be measured via Cronbach's α for the overall instrument and the individual dimensions. Cronbach's α is widely regarded a reliable and versatile coefficient, particularly applicable on Likert scale items [36].

## Results

K-S tests were conducted on each of the 35 perception/expectation scores elicited from each completion (p < 0.05). In cases where both item scores were drawn from normally distributed data, correlation was calculated via Pearson's "r" (see Table 1).

Instances featuring one or both items being drawn from non-normally distributed data, correlation calculations were made via Spearman's rho (see Table 2). Significant, positive correlations are demonstrated for all of the items calculated using Pearson's "r" (mean = 0.957, SD = 0.02) and Spearman's rho (mean = 0.934, SD = 0.03). Furthermore, Cronbach's Alpha was calculated for all 7 dimensions (35 items) at 0.903 and subsequently for each individual dimension to demonstrate internal consistency (see Table 3). Internal consistency was calculated of the second of the two completions of REFERQUAL.

The reliability and GP dimensions score particularly highly at 0.802 and 0.857 respectively. Most others scored satisfactorily; however, the tangibles and responsiveness dimensions were a little low at 0.554 and 0.619 respectively.

## Discussion

The overwhelmingly supportive statistical analysis above combined with the feedback received from the respondents and scheme organisers confirmed REFERQUAL's applicability and suitability for further evaluation of service quality in the ERS setting. The high correlation scores generated across all 35 items are especially encouraging that REFERQUAL is a valid and reliable research instrument in this setting explaining, at worst, 74% of the variance.

The dimensional reliability scores are also heartening, especially considering the overall instrument internal consistency (α = 0.903). The two dimensions scoring slightly lower than the others (responsiveness and tangibles) are not considered to give cause for concern. Responsiveness is widely acknowledged to be a fundamental contributor to the understanding and assessment of service quality [7,37] and REFERQUAL's modifications contain no new items that may have been responsible for this abnormality. Further investigations utilising this tool with much larger samples than this small-scale pilot should result in a more satisfactory report for this dimension.

However, the applicability of tangibles as a dimension within service quality assessment has received criticism, relating to client's perception of the concept as a proxy for evaluating service outcomes [37]. However, tangibles is generally retained in factor analysis [38] and the relative importance of the this dimension is thoroughly discussed within the literature, resulting in general affirmative agreement [12]. However, tangibles are the least critical of the proposed service quality dimensions [25]. Cleanliness, modern equipment and aesthetic appeal will rarely countervail poor quality products, unhelpful information and impolite staff. Future studies should incorporate factor analysis to establish whether tangibles remain distinct from the other 6 or whether this dimension requires further revision or augmentation.

Calculations within this investigation have been made via the perception/expectation score generated by the SERVQUAL format. Future studies should analyse both perception and expectation items separately to confirm the validity and reliability of this model particularly bearing in mind the fundamental advantage of the perception/expectation 'gap' model over the performance-only instruments – that the perceptions of those clients not visiting the referral sites at all could be gathered. This investigation solely sought the views of active exercises at various stages of completion of referral. Future studies should seek to investigation across the entire A-E spectrum outlined above.

## Conclusion

Preliminary results indicate REFERQUAL to represent a promising model of service quality assessment within the ERS setting. Correlational findings are extremely supportive and are underlined by satisfactory reliability scores. However, future studies incorporating the views of the entire adherence spectrum featuring far greater sample sizes and subsequent factor analysis on the dimensional structure of REFERQUAL will offer far greater insight into the appropriability of this new model.

## Declaration of competing interests

The author(s) declare that they have no competing interests.

## Authors' contributions

DC: Responsible for the overall study design and the acquisition and analysis of the data. DC was also responsible for the drafting of the document.

IA: Made substantial contributions to the conceptual design of the study and responsible for considerable drafting input.

AI: Made substantial contributions to the conceptual design of the study and responsible for considerable drafting input. Also responsible for statistical guidance and advice.

PB: Made substantial contributions to the conceptual design of the study and responsible for considerable drafting input.

## Pre-publication history

The pre-publication history for this paper can be accessed here:



## Supplementary Material

Additional File 1 Figure 1, The SERVQUAL Instrument. Click here for file

Additional File 2 Figure 2, Supplementary Questions Added to SERVQUAL. Click here for file

## Figures and Tables

**Table 1:** Pearson Correlation of Normally Distributed Items

Item	Pearson Correlation (r)	N
Responsiveness11	0.963	27
Responsiveness13	0.976	27
Assurance15	0.933	27
Empathy22	0.958	27
Empathy24	0.952	26
GP30	0.964	25
GP31	0.985	26
Personal32	0.920	27
Personal34	0.962	27

**Table 2:** Spearman's rho of Non-Normally Distributed Items

Item	Spearman Correl. (rho)	N
Tangibles 1	0.910	27
Tangibles 2	0.914	27
Tangibles 3	0.931	27
Tangibles 4	0.932	26
Responsiveness 5	0.952	27
Responsiveness 6	0.909	27
Responsiveness 7	0.917	27
Responsiveness 8	0.952	27
Responsiveness 9	0.883	25
Responsiveness 10	0.857	26
Responsiveness 12	0.950	27
Assurance 14	0.864	26
Assurance 16	0.932	27
Assurance 17	0.932	21
Assurance 18	0.972	20
Assurance 19	0.958	26
Assurance 20	0.922	27
Assurance 21	0.954	27
Empathy 23	0.924	25
Empathy 25	0.952	26
Empathy 26	0.976	25
Empathy 27	0.966	26
GP 28	0.953	26
GP 29	0.938	23
Personal 33	0.964	27
Personal 35	0.965	27

**Table 3:** Dimensional Cronbach's Alpha

Dimension	α
Tangibles	0.554
Reliability	0.802
Responsiveness	0.619
Assurance	0.678
Empathy	0.659
GP	0.857
Personal	0.644
