# Supplementary material for: REFERQUAL: a pilot study of a new service quality assessment instrument in the GP exercise referral scheme setting
Source: BMC Health Serv Res. 2006 May 25;6:61. doi: 10.1186/1472-6963-6-61 (PMC1482701; doi:10.1186/1472-6963-6-61)
Supplement: Additional File 1 — Figure 1, The SERVQUAL Instrument. [file 1472-6963-6-61-S1.doc]

Figure 1: The SERVQUAL Instrument

**DIRECTIONS:** This survey deals with your opinions of __________ services. Please show the extent to which you think firms offering _________ services should possess the features described by each statement. Do this by picking one of the seven numbers next to each statement. If you strongly agree that these firms should posses a feature, circle the number 7. If you strongly disagree that these firms should possess a feature, circle 1. If your feelings are not strong, circle one of the numbers in the middle. There are no right or wrong answers – all we are interested in is a number that best shows your expectations about the firms offering ________ services.

E1. They should have up-to-date equipment.

E2. Their physical facilities should be visually appealing.

E3. Their employees should be well dressed and appear neat.

E4. The appearance of the physical facilities of these firms should be in keeping with the type of services provided.

E5. When these firms promise to do something by a certain time, they should do so.

E6. When customers have problems, these firms should be sympathetic and reassuring.

E7. These firms should be dependable

E8. They should provide their services at the time they promise to do so.

E9. They should keep their records accurately.

E10. They shouldn’t be expected to tell customers exactly when services will be performed.

E11. It is not realistic for customers to expect prompt service from employees of these firms.

E12. Their employees don’t always have to be willing to help customers.

E13. It is okay if they are too busy to respond to customer requests promptly.

E14. Customers should be able to trust employees of these firms.

E15. Customers should be able to feel safe in their transactions with these firms’ employees.

E16. Their employees should be polite.

E17. Their employees should get adequate support from these firms to do their jobs well.

E18. These firms should not be expected to give customers individual attention.

E19. Employees of these firms cannot be expected to give customers personal attention.

E20. It is unrealistic to expect employees to know that the needs of their customers are.

E21. It is unrealistic to expect these firms to have their customers’ best interests at heart.

E22. They shouldn’t be expected to have operating hours convenient to all their customers.

**DIRECTIONS:** The following set of statements relate to your feelings about XYZ. For each statement, please show the extent to which you believe XYZ has the feature described by the statement. Once again, circling a 7 means that you strongly agree that XYZ has that feature, and circling a 1 means that you strongly disagree. You may circle any of the numbers in the middle that show how strong your feelings are. There are no right or wrong answers – all we are interested in is a number that best shows your perceptions about XYZ.

P1. XYZ has up-to-date equipment.

P2. XYZ’s physical facilities are visually appealing.

P3. XYZ’s employees are well dressed and appear neat

P4. The appearance of the physical facilities of XYZ is in keeping with the type of services provided.

P5. When XYZ promises to do something by a certain time, it does so.

P6. When you have problems, XYZ is sympathetic and reassuring.

P7. XYZ is dependable

P8. XYZ provides its services at the time it promises to do so.

P9. XYZ keeps its records accurately.

P10. XYZ does not tell customers exactly when services will be performed.

P11. You do not receive prompt service from XYZ’s employees

P12. Employees of XYZ are not always willing to help customers.

P13. Employees of XYZ are too busy to respond to customer requests promptly.

P14. You can trust employees of XYZ.

P15. You feel safe in your transactions with XYZ’s employees.

P16. Employees of XYZ are polite.

P17. Employees get adequate support from XYZ to do their jobs well.

P18. XYZ does not give you individual attention.

P19. Employees of XYZ do not give you personal attention.

P20. Employees of XYZ do not know what your needs are.

P21. XYZ does not have your best interests are heart.

P22. XYZ does not have operating hours convenient to all their customers.
