# Supplementary material for: REFERQUAL: a pilot study of a new service quality assessment instrument in the GP exercise referral scheme setting
Source: BMC Health Serv Res. 2006 May 25;6:61. doi: 10.1186/1472-6963-6-61 (PMC1482701; doi:10.1186/1472-6963-6-61)
Supplement: Additional File 2 — Figure 2, Supplementary Questions Added to SERVQUAL. [file 1472-6963-6-61-S2.doc]

Figure 2: Supplementary Questions Added to SERVQUAL

| **No.** | **Wording** | **Dimension** |
| --- | --- | --- |
| 5 | The changing rooms at the centre should be clean and well maintained | Tangibles |
| 17 | People should receive praise from the staff of these leisure centres | Assurance |
| 20 | Staff should contact people who do not attend or stop attending an Exercise Referral Scheme | Assurance |
| 21 | Exercise Professionals dealing with people on the scheme should be of a similar age to the exercisers | Assurance |
| 22 | Exercise Professionals should be able to demonstrate considerable knowledge of the benefits to health of exercise | Assurance |
| 23 | Employees of these centres should offer help and support when requested by people | Assurance |
| 26 | Employees of these centres should seek to identify people’s previous exercise experience | Empathy |
| 30 | Members of the scheme should be partnered with another person to help each other through the Exercise Referral process | Empathy |
| 31 | GPs should be enthusiastic towards the Exercise Referral procedure | GPs |
| 32 | GPs referring to an Exercise Referral program should be active themselves | GPs |
| 33 | GPs should be able to demonstrate understanding of the benefits of exercise | GPs |
| 34 | GPs should encourage doing exercise all the time | GPs |
| 35 | People have time to exercise if they want to | Personal |
| 36 | Exercise is not painful | Personal |
| 37 | Exercising is not an expensive pastime | Personal |
| 38 | Exercise is enjoyable | Personal |
